# Dietary Inflammatory Potential and Continence‐Related Symptoms: Cross‐Sectional and Longitudinal Analyses of Three Population Studies

**DOI:** 10.1002/fsn3.72477

**Published:** 2026-10-09

**Authors:** Mengmeng Jiang, Mengshi Zhong, Juan Yu, Jialing Zhang, Honggang Wang

**Affiliations:** ^1^ Department of Gastroenterology The Affiliated Huaian No. 1 People's Hospital of Nanjing Medical University Huai'an China

**Keywords:** dietary assessment, dietary inflammatory index, fecal incontinence, longitudinal study, nutritional epidemiology, older adults

## Abstract

Dietary inflammatory potential has been associated with prevalent continence problems, but cross‐population comparability and prospective associations remain uncertain. We examined these questions in three population studies. Repeated cross‐sectional analyses of the National Health and Nutrition Examination Survey (NHANES) 2005–2010 related a 25‐component Dietary Inflammatory Index (DII) to monthly mucus, liquid, or solid leakage. A 15‐component DII enabled a partially harmonized comparison with the English Longitudinal Study of Aging (ELSA). The Chinese Longitudinal Healthy Longevity Survey (CLHLS) 2008–2018 used a 13‐food directional score and combined urinary and bowel continence impairment. Analyses included logistic regression, splines, repeated measures, and sensitivity checks for dietary measurement, weighting, and missing covariates. Among 13,385 NHANES participants, each DII unit was associated with prevalent leakage (odds ratio 1.075, 95% confidence interval 1.029–1.124), with similar estimates across survey periods. Concurrent estimates were 1.136 (1.066–1.210) per 15‐component DII unit in ELSA and 1.440 (1.135–1.828) per score standard deviation in CLHLS. Combined weighting in ELSA and cognitive adjustment in CLHLS attenuated the estimates, with intervals including the null. Cumulative incident estimates were 0.974 (0.902–1.051) in ELSA and 1.044 (0.920–1.184) in CLHLS, using the respective exposure units. Concurrent associations were observed in the main analyses, but baseline scores showed no clear associations with incident outcomes. Dietary measurement differences, symptom‐related dietary changes, and functional or socioeconomic confounding limit interpretation. These findings extend cross‐sectional evidence without establishing that lowering dietary inflammatory potential prevents continence‐related symptoms.

## Graphical abstract

Dietary inflammatory potential and continence‐related symptoms across NHANES, ELSA, and CLHLS. Higher dietary scores were associated with existing symptoms in the main analyses, whereas prospective analyses showed no clear associations with incident outcomes. Differences in populations, dietary measures, and outcomes limit direct comparison. These observational findings do not establish causality or preventive benefit from dietary modification. The graphical abstract and its illustrative icons were generated with Image 2 (OpenAI) during manuscript revision, based on author‐directed descriptions of the study. The authors reviewed and verified the content.

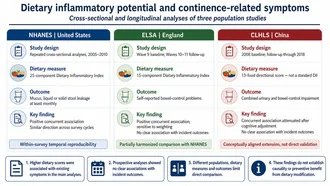

## 1 Introduction

Normal stool formation and passage depend on colonic water absorption and coordinated propulsion towards the rectum (Heitmann et al. 2021). The physical properties of dietary fiber, including water‐holding and gel‐forming capacity, influence stool water content and consistency (McRorie Jr. and McKeown 2017). Rectal storage and sensation, together with anal sphincter and pelvic floor function, maintain continence until coordinated propulsion and outlet relaxation permit defecation (Bharucha et al. 2022; Heitmann et al. 2021). Fecal incontinence (FI) is the involuntary loss of liquid or solid stool. Diarrhea, constipation with overflow, pelvic floor injury, neurological disease, and functional limitations can contribute (Assmann et al. 2022; Bharucha et al. 2022). Leakage can restrict social activities and eating outside the home, while embarrassment may discourage disclosure (Bharucha et al. 2022). Diet is relevant both as a possible contributor to bowel symptoms and as a behavior that may change in response to them.

Fiber properties, fermentable carbohydrates, and individual food tolerance may influence stool water, bulk, gas production, and transit. Symptom‐driven food avoidance can also alter dietary intake (Andy et al. 2020; Bliss et al. 2014; Menees et al. 2022). The Dietary Inflammatory Index (DII) summarizes dietary inflammatory potential using literature‐derived component scores (Shivappa et al. 2014). It measures a dietary construct rather than intestinal inflammation or anorectal function. Associations with systemic inflammatory markers do not establish an inflammatory mechanism for continence‐related symptoms (Mohammadi et al. 2023). A prospective study in older women reported an association between pro‐inflammatory diet and FI (Yang et al. 2023). Two National Health and Nutrition Examination Survey (NHANES) analyses also reported cross‐sectional associations (Li, Chen, et al. 2024; Wang et al. 2025). Whether these associations extend across dietary measures, population contexts, and subsequent symptoms remains unresolved.

We addressed five questions: whether the dietary association with existing symptoms was nonlinear; whether it was reproducible across NHANES survey periods; whether a partially harmonized comparison in the English Longitudinal Study of Aging (ELSA) supported a similar concurrent association; whether baseline dietary scores were associated with incident symptoms in ELSA and the Chinese Longitudinal Healthy Longevity Survey (CLHLS); and what a conceptually aligned CLHLS extension added. NHANES and ELSA were compared using a common 15‐component score. CLHLS used a different dietary score and a combined urinary/bowel outcome and was not a direct validation. The incident analyses excluded participants with the respective baseline outcome. NHANES cycle overlap with previous studies precludes independent US population replication.

## 2 Materials and Methods

### 2.1 Design and Data Sources

We conducted repeated cross‐sectional and longitudinal secondary analyses of three population studies and followed the Strengthening the Reporting of Observational Studies in Epidemiology (STROBE) reporting framework (von Elm et al. 2007). NHANES uses a stratified multistage probability design to represent the non‐institutionalized United States population. The primary analysis used the 2005–2006, 2007–2008, and 2009–2010 periods. ELSA is a panel study of adults aged 50 years or older in England that also includes younger partners; Wave 9 dietary data were linked with Waves 10 and 11 (Steptoe et al. 2013). CLHLS followed older adults across 23 provincial‐level areas in China; the 2008 cohort was linked with the 2011, 2014, and 2018 waves (Center for Healthy Aging and Development Studies 2020). The required bowel‐health questionnaire items were available in the 2005–2010 NHANES cycles, which determined the period selected for analysis (National Center for Health Statistics 2007). Figure 1 summarizes study periods, measurement definitions, and analytic roles. Table S1 compares the present analyses with the two previous NHANES studies. Table S2 details population characteristics and differences in dietary instruments and outcomes.

**FIGURE 1:**
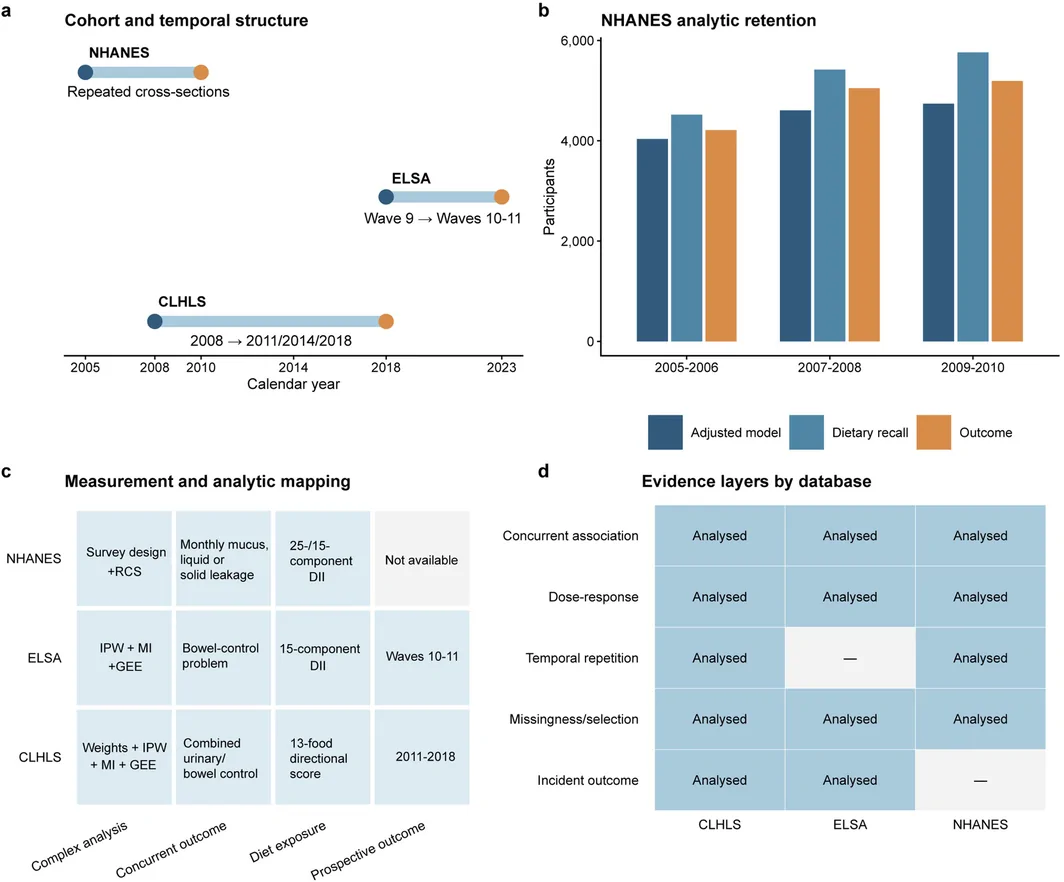
Study design, measurement definitions and analytic roles across three population studies. (a) NHANES survey periods and the ELSA and CLHLS follow‐up structure. (b) NHANES participant counts with dietary recalls, valid outcomes and complete covariates for the adjusted model, by survey period. (c) Analytic approaches, concurrent outcomes, dietary exposures and prospective outcome waves. (d) Types of analyses available within each database. The matrix indicates analyses performed, not equivalent measurements or strength of evidence. Within‐NHANES temporal reproducibility is distinguished from the partially harmonized ELSA comparison and conceptually aligned CLHLS extension. Detailed age ranges and dietary instruments are reported in Table S2. CLHLS, Chinese Longitudinal Healthy Longevity Survey; DII, Dietary Inflammatory Index; ELSA, English Longitudinal Study of Aging; GEE, generalized estimating equation; IPW, inverse‐probability weighting; MI, multiple imputation; NHANES, National Health and Nutrition Examination Survey; RCS, restricted cubic spline.

### 2.2 Participants

The NHANES analysis included adults aged at least 20 years with a reliable first‐day dietary recall, positive dietary sampling weight and valid FI items. The primary complete‐case model additionally required age, sex, race and ethnicity, education, marital status, family income‐to‐poverty ratio and smoking information. The ELSA concurrent analysis included Wave 9 nutrition participants, including eligible younger partners, with a reconstructed 15‐component score and valid bowel‐control outcome. An additional sensitivity analysis restricted age to at least 50 years. Prospective analyses were restricted to participants without a bowel‐control problem at baseline and with an eligible follow‐up response. The CLHLS baseline sample was restricted to participants aged at least 65 years in 2008. Prospective analyses were restricted to participants reporting complete continence control at baseline.

### 2.3 Dietary Inflammatory Exposure in NHANES

The original DII was reconstructed using the published scoring framework (Shivappa et al. 2014). The 25 consistently available components were alcohol, vitamins B12 and B6, beta‐carotene, caffeine, carbohydrate, cholesterol, energy, total fat, fiber, folic acid, iron, magnesium, monounsaturated fat, niacin, protein, polyunsaturated fat, thiamine, riboflavin, saturated fat, selenium, zinc, vitamins A, C and E. For each component, intake was standardized against the published global mean and standard deviation, mapped through the standard normal cumulative distribution to a centered percentile and multiplied by its literature‐derived inflammatory effect score. Component scores were summed; higher scores represented greater pro‐inflammatory potential. The primary analysis used the first 24‐h recall. A sensitivity analysis averaged both recall days among participants with two recalls before calculating DII. The energy‐covariate sensitivity analysis removed energy from the score (24 components) and added energy intake to the regression; it was not the standard energy‐density E‐DII. Plausible intake was defined as 500–3500 kcal/day for women and 800–4200 kcal/day for men, inclusive. Folic acid used DR1TFA rather than total folate; caffeine was converted from mg to g, and vitamin A used historical retinol equivalents. The component dictionary gives source variables, conversions and scoring parameters. A common 15‐component DII, limited to components also available in ELSA, was calculated separately for cross‐database comparison.

### 2.4 Fecal Incontinence Outcomes in NHANES

The primary NHANES survey outcome was at least monthly leakage of mucus, liquid stool or solid stool during the previous 30 days, based on BHQ020, BHQ030 and BHQ040. All three items had to have valid codes 1–6; any code 1–5 indicated leakage and three code‐6 responses indicated no leakage. Code 5 denotes one to three times per month. Gas alone was excluded. Thus, this operational survey definition includes mucus‐only cases and is broader than a strict liquid‐or‐solid‐stool definition of FI. Separate analyses considered each of the three leakage types, with Holm correction across the comparisons. The Fecal Incontinence Severity Index was analyzed among primary‐outcome cases with valid severity data (Rockwood et al. 1999).

### 2.5 ELSA Exposure and Outcomes

Wave 9 intake was derived from the Oxford WebQ online 24‐h dietary questionnaire and the participant‐level means across available dietary days (Liu et al. 2011; UK Data Service 2020). A 2‐day sensitivity analysis retained exactly two recorded days. The comparison score used alcohol, vitamins B12 and B6, carotene, carbohydrate, energy, total fat, fiber, iron, magnesium, protein, polyunsaturated fat, saturated fat, and vitamins C and E. The supplied component dictionary specifies each source variable, unit, conversion, reference mean and standard deviation, and effect score. ELSA mean carotene was mapped to the beta‐carotene scoring parameter in the original implementation; the available label does not establish exact equivalence to NHANES beta‐carotene. We therefore also fitted 14‐component scores excluding this term in both cohorts. The comparison remains only partially harmonized. The concurrent outcome was a self‐reported bowel‐control problem during the preceding 12 months; the persistence outcome required a reported duration exceeding 1 month. Incident outcomes were new problems at Wave 10, Wave 11 or either follow‐up among participants without the problem at Wave 9. Instruments, recall windows, and outcomes were not calibrated to equivalence.

### 2.6 CLHLS Exposure and Outcome

A 13‐food directional score was constructed from 13 current food‐frequency items: fresh fruit, vegetables, meat, fish, eggs, legumes, salt‐preserved vegetables, sugar, tea, garlic, dairy products, nuts, and mushrooms or algae. Infrequent consumption of fruit, vegetables, fish, eggs, legumes, tea, garlic, dairy products, nuts, and mushrooms or algae contributed to a higher score; frequent consumption of meat, salt‐preserved vegetables, and sugar contributed to a higher score. Components were scaled from zero to one and averaged when at least 11 of 13 were valid. The score was standardized using the 2008 mean and standard deviation. This measure was not a standard DII and was not directly compared numerically with the nutrient‐based DII. An alternative 10‐item score excluded eggs, dairy products, and tea because their assigned direction was potentially contestable. CLHLS item E5 asked whether the respondent could control urination and defecation. Responses distinguished complete control, occasional accidents, and catheter assistance or inability to control. The primary outcome combined the latter two as continence impairment; the strict outcome retained only catheter assistance or inability to control. Because the item combined urinary and bowel control, it was not labeled FI. The food directions were scoring assumptions; their biological interpretation and the alternative score are discussed in Section 4.5. The score has not been validated as a measure of inflammation in this analysis.

### 2.7 Covariates

NHANES covariates included age, sex, race and ethnicity, education, marital status, family income‐to‐poverty ratio and smoking. Extended models added body mass index, diabetes and depressive symptoms. ELSA models adjusted for age, sex, ethnicity, age at leaving full‐time education, marital status (married, remarried, or in a registered civil partnership versus other recorded categories) and current smoking; sensitivity models additionally considered physical activity, diabetes, stroke, cancer and depressive symptoms. CLHLS models adjusted for age, sex, ethnicity, urban residence, years of education, marital status, relative economic status, smoking, drinking and exercise. Cognition and body mass index were examined as extended adjustments.

### 2.8 Statistical Analysis

NHANES logistic models incorporated strata, primary sampling units, and dietary weights, using WTDRD1/3 for day‐1 analyses and WTDR2D/3 for two‐day analyses. Odds ratios (ORs) used survey‐design variance; coefficient confidence intervals (CIs) and *p* values used a normal Wald approximation. Restricted cubic splines used weighted percentile knots and a weighted median reference (Desquilbet and Mariotti 2010). Design‐based tests assessed overall association and nonlinearity; period‐specific estimates were compared using fixed‐effect heterogeneity statistics. Holm correction covered the three leakage‐type comparisons. ELSA primary models were unweighted because nutrition‐substudy‐specific weights were unavailable. The core Wave 9 weight was examined separately and with nutrition‐participation inverse‐probability weighting (IPW). Participation and attrition IPWs address observed selection rather than reconstruct nutrition‐specific sampling or calibration weights. CLHLS models retained baseline sampling weights. ELSA and CLHLS weighted logistic models used fixed‐weight, independent‐person sandwich covariance; CLHLS sampling clusters and strata were not reconstructed. Their spline analyses used unweighted percentile knots, with likelihood‐ratio nonlinearity testing in ELSA and robust Wald testing in CLHLS. Twenty stochastic hot‐deck imputations of missing covariates were pooled using Rubin's rules and t‐based intervals. Generalized estimating equations (GEEs) used a binomial logit link, independence working correlation, and participant‐clustered robust variance. Supporting Information Methods specify knot locations, selection predictors, weight truncation, imputation donors, and exposure timing. Age‐restricted and 14‐component analyses were added during revision to assess the ELSA age range and uncertain carotene mapping. Tests were two‐sided, using R 4.6.1, survey 4.5, and Python statsmodels 0.15.0. Interpretation considered effect sizes, uncertainty, and temporal ordering rather than statistical significance alone.

## 3 Results

### 3.1 NHANES Sample and Primary Association

Across the three periods, 15,702 adults had a reliable first‐day dietary recall and 14,454 had complete primary outcomes; 1328 reported leakage. The primary complete‐case model included 13,385 participants and 1213 events. Weighted prevalence in the outcome‐complete sample was 8.45% (95% CI 7.71%–9.18%). Each one‐unit higher DII was associated with the primary outcome in the core‐adjusted model (OR 1.075 (95% CI 1.029–1.124); *p* = 0.001). The extended model additionally adjusting for body mass index, diabetes and depressive symptoms yielded OR 1.056 (95% CI 1.009–1.106) (*p* = 0.020). The quartile‐4 versus quartile‐1 OR was 1.514 (95% CI 1.171–1.957). The overall spline test was *p* = 0.025 and the nonlinearity test was *p* = 0.954, providing no evidence of nonlinearity within the fitted model (Figure 2). Table 1 reports primary and period‐specific estimates.

**FIGURE 2:**
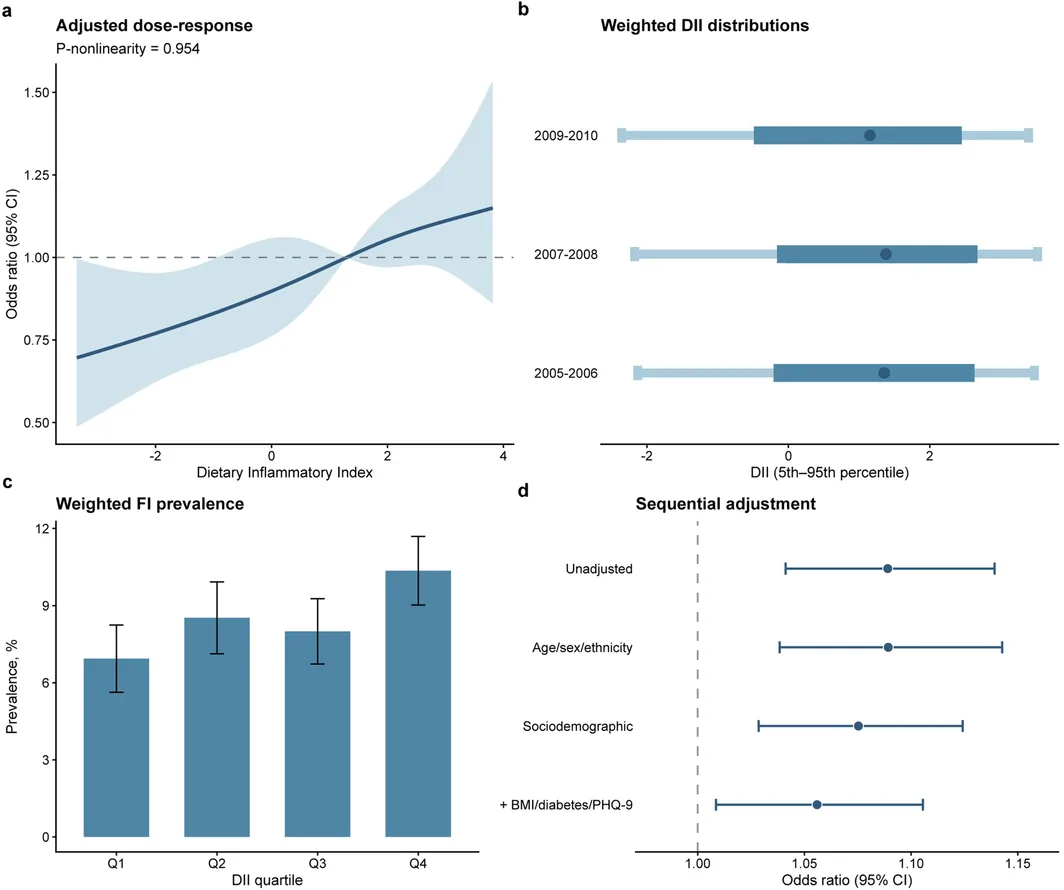
Primary NHANES dose–response evidence. (a) Core‐adjusted restricted cubic spline for DII and the primary leakage outcome; the weighted median is the reference, and shading denotes the 95% confidence interval. The nonlinearity test was *p* = 0.954. (b) Survey‐weighted DII distributions by survey period. Points indicate medians, thick bars indicate the 25th–75th percentiles, and outer intervals indicate the 5th–95th percentiles. These distribution intervals are not confidence intervals. (c) Survey‐weighted prevalence across DII quartiles, with 95% confidence intervals. (d) Sequential regression models from unadjusted to extended adjustment; points and horizontal bars indicate odds ratios and 95% confidence intervals per one‐unit higher DII. The operational outcome includes at least monthly mucus, liquid‐stool, or solid‐stool leakage. Dashed reference lines indicate an odds ratio of 1. BMI, body mass index; CI, confidence interval; DII, Dietary Inflammatory Index; FI, fecal incontinence; NHANES, National Health and Nutrition Examination Survey; PHQ‐9, nine‐item Patient Health Questionnaire; Q, quartile.

**TABLE 1:** Main and temporally repeated NHANES associations.

Analysis	*N*/events	OR	95% CI	*p*
Main DII, core adjusted	13,385/1213	1.075	1.029–1.124	0.001
2005–2006	4037/332	1.081	0.974–1.200	0.144
2007–2008	4607/417	1.071	1.021–1.124	0.005
2009–2010	4741/464	1.071	1.012–1.133	0.018
Leave‐one‐period‐out range	—	1.072–1.077	—	—

*Note:* ORs are per one‐unit higher 25‐component DII. Dashes indicate values not reported in this summary; they are not zero. *N*/events denotes analyzed participants/outcome events.

Abbreviations: CI, confidence interval; CLHLS, Chinese Longitudinal Healthy Longevity Survey; DII, Dietary Inflammatory Index; ELSA, English Longitudinal Study of Aging; FI, fecal incontinence; IPW, inverse‐probability weighting; NHANES, National Health and Nutrition Examination Survey; OR, odds ratio.

### 3.2 Temporal Reproducibility and Sensitivity Analyses

Core‐adjusted ORs per one‐unit higher DII were 1.081 (95% CI 0.974–1.200) in 2005–2006, 1.071 (95% CI 1.021–1.124) in 2007–2008, 1.071 (95% CI 1.012–1.133) in 2009–2010. There was no evidence of heterogeneity (interaction *p* = 0.995; *I* ^2^ = 0%). Leave‐one‐period‐out ORs ranged from 1.072 to 1.077. The two‐day mean analysis yielded OR 1.075 (95% CI 1.022–1.131); plausible‐energy restriction, 1.087 (95% CI 1.036–1.141); and the 24‐component score plus energy covariate, 1.143 (95% CI 1.075–1.215). Liquid‐stool leakage remained associated after Holm correction (OR 1.093 (95% CI 1.039–1.151); adjusted *p* = 0.002). Evidence for mucus and solid‐stool leakage was insufficient after correction. The severity association was imprecise (beta 0.257, 95% CI −0.021 to 0.536; *p* = 0.070; *n* = 1212). Figure 3 presents period‐specific estimates, dietary and leakage‐type sensitivity analyses, leave‐one‐period‐out estimates, and recall‐score correlations.

**FIGURE 3:**
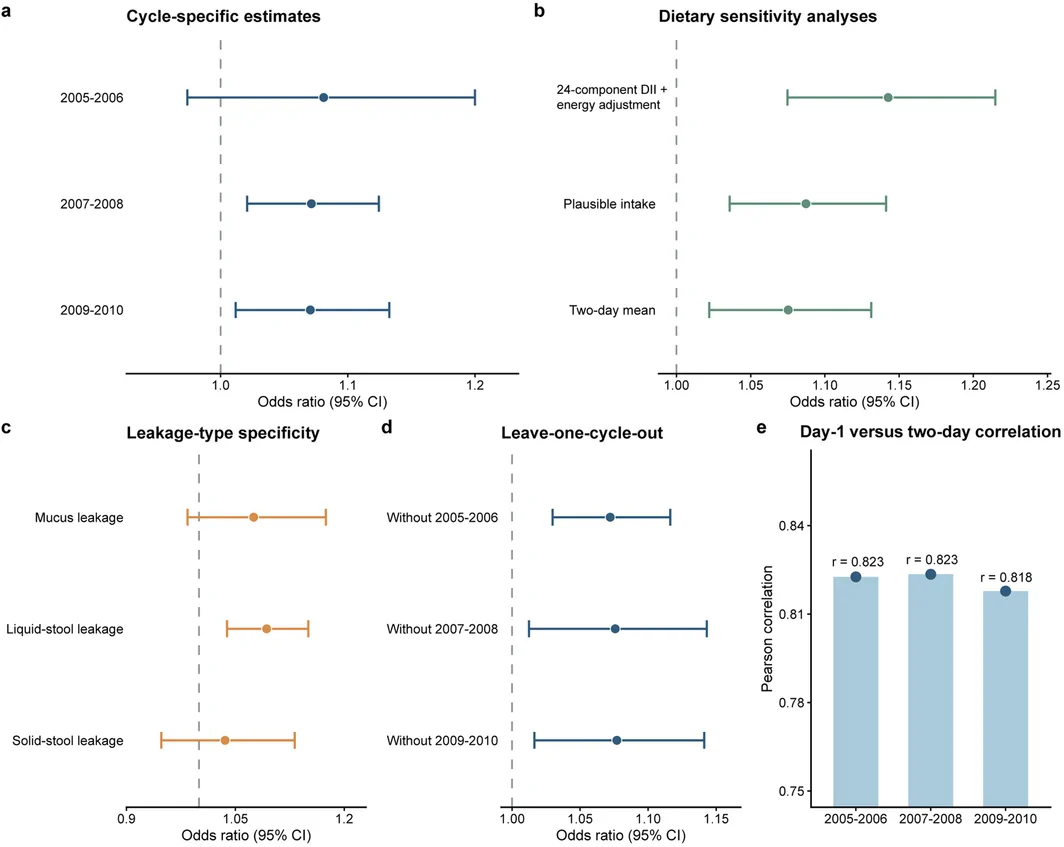
NHANES temporal reproducibility and sensitivity analyses. (a) Core‐adjusted estimates by survey period. (b) The 24‐component score with energy as a regression covariate, plausible‐energy restriction, and two2‐day recall mean, from top to bottom. The first analysis is not standard energy‐density E‐DII. (c) Separate mucus, liquid‐stool, and solid‐stool leakage analyses, from top to bottom. Confidence intervals are pointwise; Holm‐adjusted *p* values across the three comparisons are reported in the Results. (d) Leave‐one‐period‐out estimates. (e) Pearson correlations between day‐1 and 2‐day‐average DII among participants with two reliable recalls. These scores share the day‐1 recall; correlation does not establish agreement or independent validation. Forest points and bars show odds ratios and 95% confidence intervals per one‐unit higher score, with dashed reference lines at 1. CI, confidence interval; DII, Dietary Inflammatory Index; NHANES, National Health and Nutrition Examination Survey.

### 3.3 Partially Harmonized Comparison With ELSA

The concurrent 15‐component comparison included 13,385 NHANES participants and 4141 ELSA participants. Adjusted ORs per score unit were 1.128 (95% CI 1.058–1.202) in NHANES and 1.136 (95% CI 1.066–1.210) in ELSA. ELSA nonlinearity *p* was 0.951; persistent symptoms yielded OR 1.183 (95% CI 1.078–1.298). Incident ORs were 0.993 (95% CI 0.902–1.094) at Wave 10, 0.972 (95% CI 0.884–1.069) at Wave 11, and 0.974 (95% CI 0.902–1.051) cumulatively. Nutrition‐selection IPW retained a concurrent association, whereas core weight multiplied by nutrition IPW was less precise (OR 1.088 (95% CI 0.998–1.187); *p* = 0.055). Imputed concurrent and cumulative incident ORs were 1.137 (95% CI 1.067–1.212) and 0.975 (95% CI 0.903–1.052), respectively. Restriction to age ≥ 50 and exclusion of carotene did not materially change the concurrent‐versus‐incident pattern (Table S3). In all‐wave GEE, the association was strongest at Wave 9; the score‐by‐wave interaction was *p* = 0.047. Incident discrete‐time GEE yielded OR 0.985 (95% CI 0.913–1.063). Figure 4, Table 2 distinguish these analyses.

**FIGURE 4:**
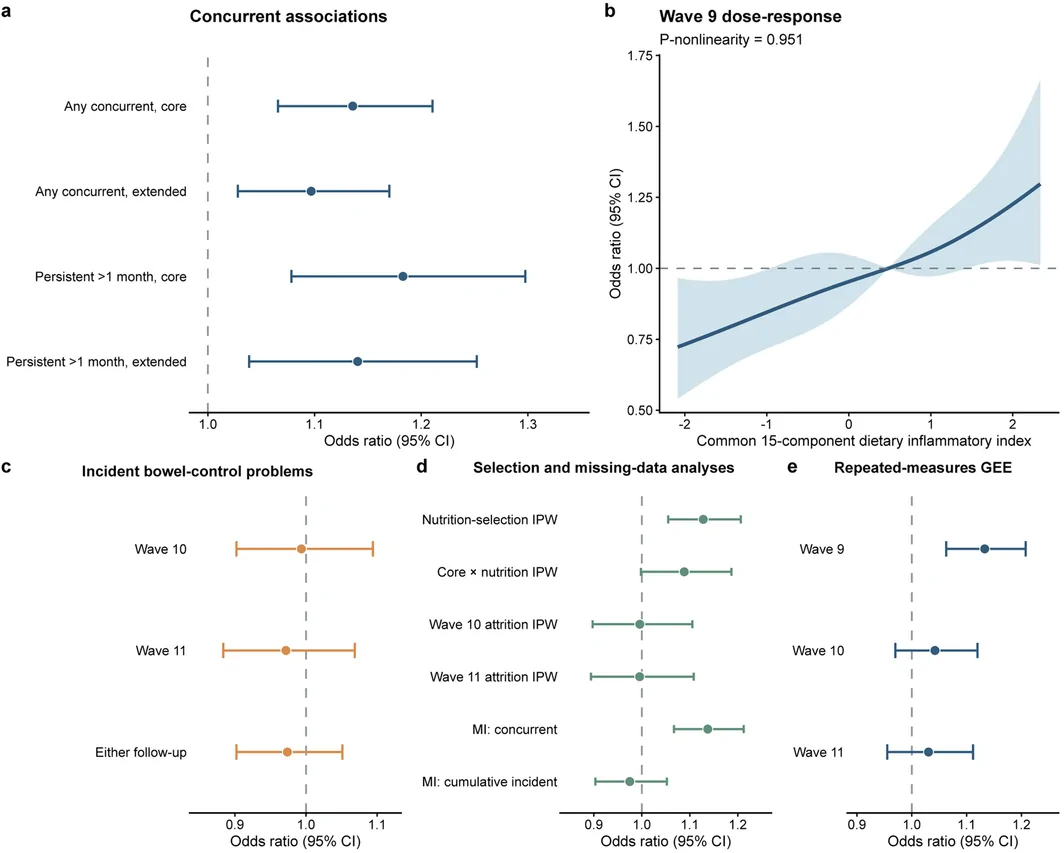
ELSA partially harmonized comparison and longitudinal findings. (a) Concurrent bowel‐control problems and symptoms persisting for more than 1 month under core and extended adjustment. (b) Wave 9 restricted cubic spline for the common 15‐component DII; the nonlinearity test was *p* = 0.951. (c) Incident bowel‐control problems at Wave 10, Wave 11 and either follow‐up among participants without the baseline outcome. (d) Nutrition‐selection IPW, core Wave 9 weight multiplied by nutrition IPW, follow‐up attrition‐IPW, and multiple‐imputation analyses. The combined core‐weight and nutrition‐IPW estimate was 1.088 (95% CI 0.998–1.187). (e) Wave‐specific repeated‐measures GEE estimates; these are distinct from the baseline‐outcome‐free incident analyses in panel c. Forest points and bars show odds ratios and 95% confidence intervals per one‐unit higher comparison score; spline shading denotes 95% confidence intervals. Dashed reference lines indicate an odds ratio of 1. Weighted sensitivity intervals treat estimated weights as fixed and do not establish design‐based population inference. CI, confidence interval; DII, Dietary Inflammatory Index; ELSA, English Longitudinal Study of Aging; GEE, generalized estimating equation; IPW, inverse‐probability weighting; MI, multiple imputation.

**TABLE 2:** Partially harmonized NHANES and ELSA results.

Database and outcome	*N*/events	OR per one‐unit DII	95% CI	*p*
NHANES concurrent leakage	13,385/1213	1.128	1.058–1.202	< 0.001
ELSA Wave 9 concurrent problem	4141/646	1.136	1.066–1.210	< 0.001
ELSA persistent problem > 1 month	4138/284	1.183	1.078–1.298	< 0.001
ELSA Wave 10 incident problem	2511/253	0.993	0.902–1.094	0.894
ELSA Wave 11 incident problem	2318/270	0.972	0.884–1.069	0.554
ELSA either follow‐up incident problem	2837/430	0.974	0.902–1.051	0.495

*Note:* ORs are per one‐unit higher 15‐component comparison score; outcome definitions differ between the cohorts. *N*/events denotes analyzed participants/outcome events. The carotene mapping is only partially aligned; Table S3 excludes this component.

Abbreviations: CI, confidence interval; CLHLS, Chinese Longitudinal Healthy Longevity Survey; DII, Dietary Inflammatory Index; ELSA, English Longitudinal Study of Aging; FI, fecal incontinence; IPW, inverse‐probability weighting; NHANES, National Health and Nutrition Examination Survey; OR, odds ratio.

### 3.4 CLHLS Conceptually Aligned Extension

The 2008 CLHLS cohort included 16,954 participants; 16,944 had a valid food score and 895 reported combined continence impairment. The core‐adjusted model included 16,110 participants and 822 events, yielding OR 1.440 (95% CI 1.135–1.828) per baseline score standard deviation (*p* = 0.003). The robust spline nonlinearity test was *p* = 0.996. The alternative 10‐item score yielded OR 1.640 (95% CI 1.282–2.098), the strict outcome 1.588 (95% CI 1.053–2.395), and additional BMI adjustment 1.408 (95% CI 1.074–1.845). Complete‐case cognitive adjustment yielded OR 1.158 (95% CI 0.880–1.523) (*p* = 0.296); 20 imputations with cognition yielded OR 1.256 (95% CI 0.987–1.597) (*p* = 0.063). Both cognition‐adjusted intervals included 1. Excluding proxy‐assisted interviews left 54 events and yielded OR 1.255 (95% CI 0.795–1.982). The cumulative incident analysis included 8021 baseline‐continent participants and 920 events. Baseline‐weighted and response‐IPW ORs were 1.044 (95% CI 0.920–1.184) and 1.029 (95% CI 0.915–1.158), respectively. The separate 2011, 2014 and 2018 incident intervals also included 1. Four‐wave GEEs produced time‐varying concurrent ORs of 1.469, 1.289, 1.144 and 1.366, respectively, with score‐by‐wave interaction *p* = 0.328. These GEE associations with contemporaneous dietary scores are not estimates of baseline diet predicting onset. Figure 5 presents retention, dose–response, sensitivity, subgroup and repeated‐measures results; Table 3 reports main CLHLS estimates.

**FIGURE 5:**
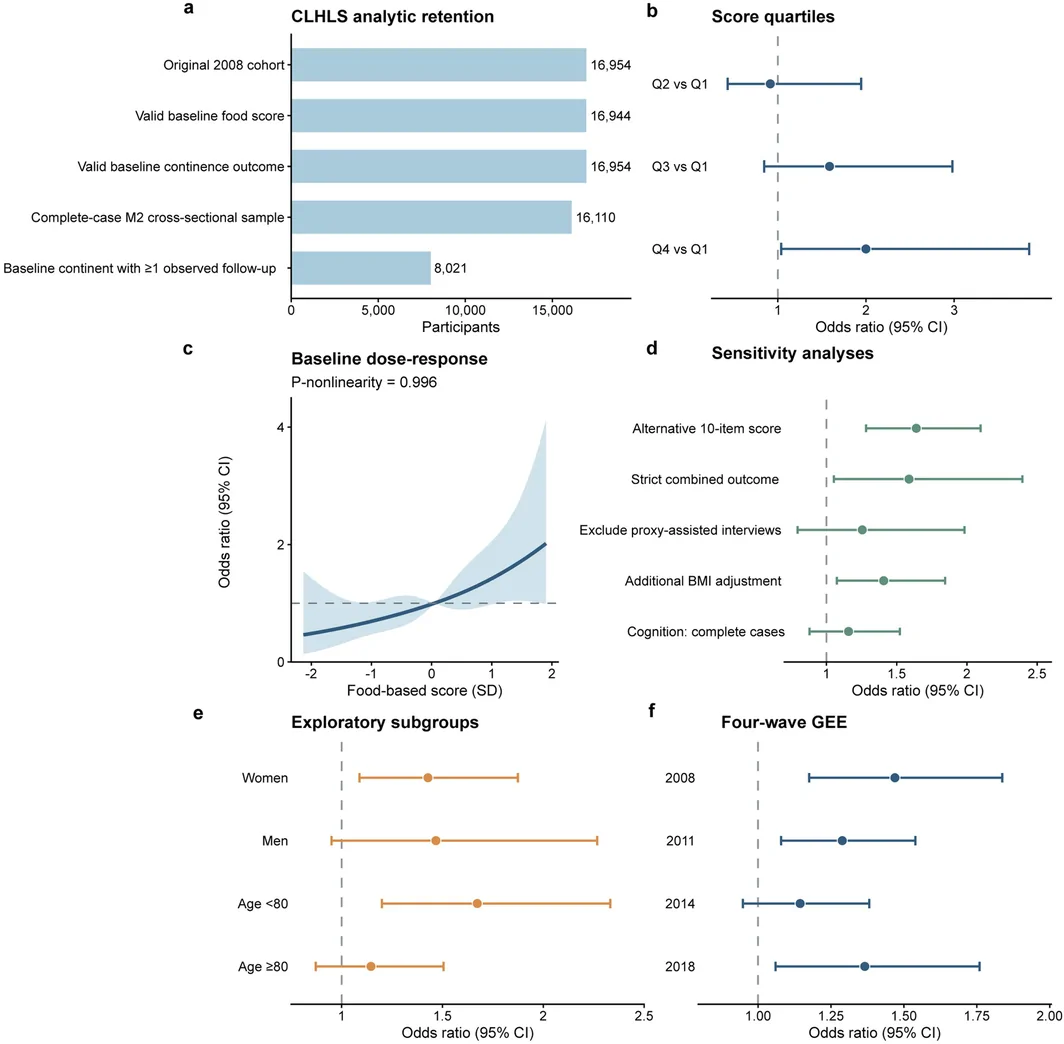
CLHLS Chinese older‐adult extension. (a) Baseline and longitudinal sample retention. (b) Weighted concurrent continence‐impairment estimates across quartiles of the food‐based score. (c) Baseline restricted cubic spline with the median as reference. (d) Alternative 10‐item score, strict combined outcome, exclusion of proxy‐assisted interviews, additional BMI adjustment and complete‐case cognitive adjustment, from top to bottom. (e) Exploratory sex‐ and age‐stratified estimates. (f) Four‐wave GEE estimates using contemporaneous dietary scores; these are not estimates of baseline diet predicting incident impairment. The exposure is a standardized, 13‐food directional score and is not a standard DII; the outcome combines urinary and bowel control. Abbreviations: DII, Dietary Inflammatory Index; CI, confidence interval; SD, standard deviation; GEE, generalized estimating equation; BMI, body mass index; Q, quartile; CLHLS, Chinese Longitudinal Healthy Longevity Survey. The strict outcome in panel d includes catheter assistance or inability to control and remains a combined urinary/bowel measure. The cognition‐adjusted estimate in panel d is the complete‐case estimate (14,668 participants; 527 events); the imputed estimate is reported in Table 3. Both cognition‐adjusted confidence intervals include 1. The spline nonlinearity test was *p* = 0.996. Panel b compares score quartiles with Q1; panels d‐f report odds ratios per one‐standard‐deviation higher score. Points and bars show odds ratios and 95% confidence intervals; spline shading denotes 95% confidence intervals. Dashed reference lines indicate an odds ratio of 1. Sampling weights are used, but variance does not reconstruct the full CLHLS sampling design.

**TABLE 3:** CLHLS conceptually aligned analyses.

Analysis	*N*/events or observations	OR per one‐standard‐deviation score	95% CI	*p*
2008 core adjusted	16,110/822	1.440	1.135–1.828	0.003
Multiple imputation plus cognition	16,199/838	1.256	0.987–1.597	0.063
Alternative 10‐item score	16,111/822	1.640	1.282–2.098	< 0.001
Strict combined outcome	16,110/186	1.588	1.053–2.395	0.027
Cumulative incident impairment	8021/920	1.044	0.920–1.184	0.503
Cumulative incident, response IPW	8021/920	1.029	0.915–1.158	0.631

*Note:* ORs are per one‐standard‐deviation higher food‐based score; the outcome combines urinary and bowel control. *N*/events denotes analyzed participants/outcome events. Weighted‐model intervals use a fixed‐weight independent‐person sandwich, not a reconstructed full survey design; the imputed interval uses Rubin pooling.

Abbreviations: CI, confidence interval; CLHLS, Chinese Longitudinal Healthy Longevity Survey; DII, Dietary Inflammatory Index; ELSA, English Longitudinal Study of Aging; FI, fecal incontinence; IPW, inverse‐probability weighting; NHANES, National Health and Nutrition Examination Survey; OR, odds ratio.

## 4 Discussion

Higher dietary scores were associated with existing continence‐related symptoms in the main analyses, but baseline scores showed no clear associations with incident outcomes. NHANES estimates were similar across survey periods and remained positive under several dietary specifications. ELSA provided a partially harmonized concurrent comparison, while cognitive adjustment attenuated the CLHLS association. The study therefore extends earlier cross‐sectional evidence by examining its reproducibility and prospective relevance across different dietary measures and population contexts. Figure 6 summarizes these findings and their implications for dietary assessment.

**FIGURE 6:**
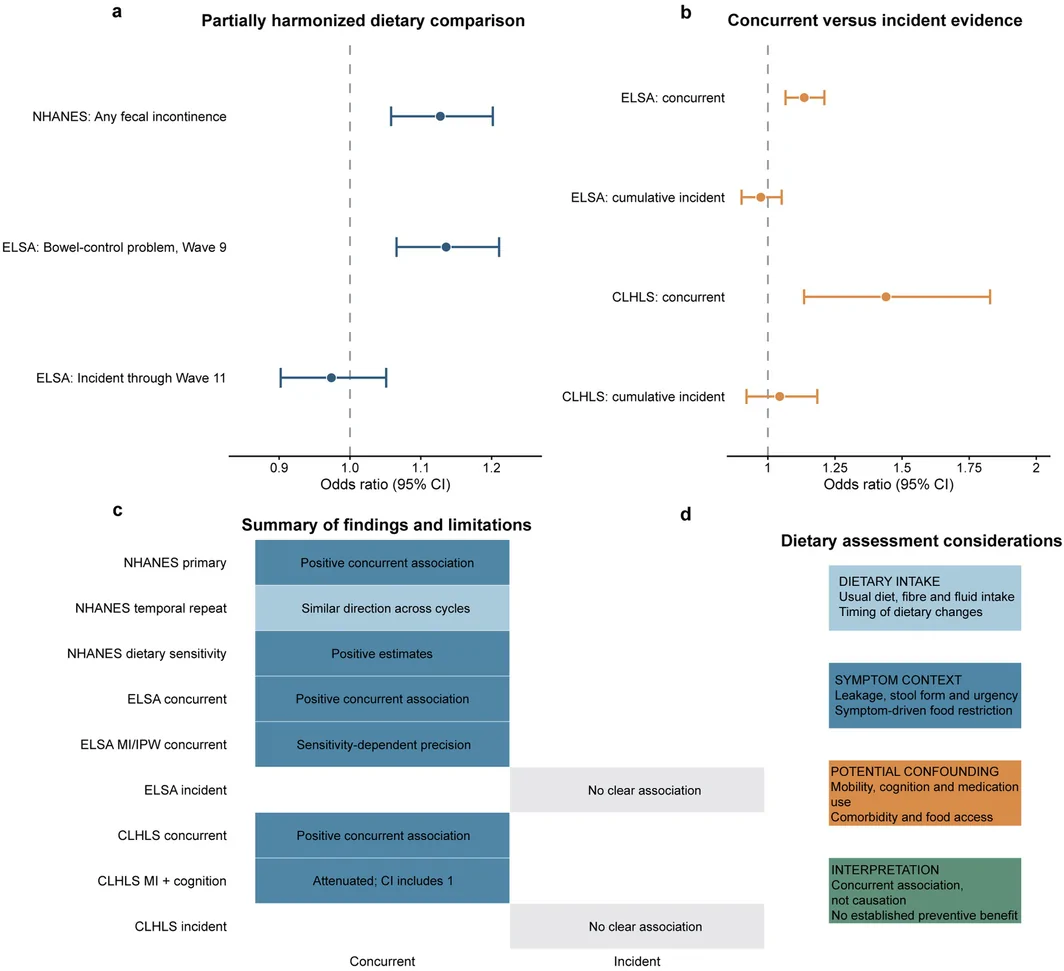
Cross‐cohort findings and dietary assessment considerations. (a) Partially harmonized comparison using the 15‐component DII in NHANES and ELSA. Estimates are per one‐unit higher score for NHANES concurrent leakage (13,385 participants; 1213 events), ELSA concurrent problems (4141/646), and ELSA cumulative incident problems (2837/430). (b) Concurrent and cumulative incident estimates within ELSA and CLHLS. ELSA estimates are per one‐unit higher 15‐component DII; CLHLS estimates are per one‐standard‐deviation higher 13‐food score (concurrent: 16,110/822; incident: 8021/920). ELSA estimates are repeated from panel a to facilitate the concurrent‐versus‐incident comparison. Points and horizontal bars show odds ratios and 95% confidence intervals; dashed lines indicate an odds ratio of 1. Different exposure scales and outcome definitions preclude pooling or ranking cohort effect sizes. (c) Summary of findings and limitations, including sensitivity‐dependent precision in ELSA and attenuation after cognitive adjustment in CLHLS. The CLHLS MI‐plus‐cognition row summarizes the imputed estimate in Table 3; Figure 5d instead shows complete‐case cognitive adjustment. No clear incident association does not establish equivalence to no effect. (d) Dietary assessment considerations covering usual intake, symptom‐related restrictions, potential confounding and interpretive limits; this is not a validated clinical pathway. Estimates are drawn from the models reported in Tables 2 and 3; no additional model was fitted for this synthesis. CI, confidence interval; CLHLS, Chinese Longitudinal Healthy Longevity Survey; DII, Dietary Inflammatory Index; ELSA, English Longitudinal Study of Aging; IPW, inverse‐probability weighting; MI, multiple imputation; NHANES, National Health and Nutrition Examination Survey.

### 4.1 Dose–Response Shape and Dietary Mechanisms

The spline analyses found no evidence of nonlinearity in NHANES (*p* = 0.954) or CLHLS (*p* = 0.996), although these findings do not establish exact linearity. Similar nonlinearity tests across dietary scores do not establish a shared biological dose–response function. Liquid‐stool leakage remained associated after correction for the three symptom‐type comparisons. This finding motivates attention to stool properties but does not establish a mechanism or a difference in effects between leakage types. Dietary components may influence continence through different processes. In a randomized trial of adults with loose or liquid fecal leakage, psyllium reduced episode frequency and formed a fecal gel. Carboxymethylcellulose increased episode frequency (Bliss et al. 2014). These findings emphasize water‐holding and gel‐forming properties rather than an equivalent effect of all fiber. A pilot trial compared a low‐FODMAP diet with psyllium in people with FI and loose stools (Menees et al. 2022). FODMAPs are fermentable oligosaccharides, disaccharides, monosaccharides, and polyols. Poorly absorbed carbohydrates can affect luminal water and fermentation, without necessarily corresponding to their contribution to an inflammatory diet score. Fat, caffeine, alcohol, and particular foods may also relate to stool consistency, transit, or urgency, with responses depending on food preparation and individual tolerance (Andy et al. 2020; Assmann et al. 2022). These symptom‐management pathways differ from the broader systemic‐inflammatory construct represented by DII. Fiber is only one component, and a higher total score does not necessarily indicate low fiber intake in every participant. We did not test mediation through inflammatory biomarkers, fermentation products, transit, or anorectal physiology. An inflammatory mechanism remains one hypothesis alongside effects on stool properties, reverse causation, and residual confounding. Trials addressing established symptoms cannot demonstrate prevention through lowering DII.

### 4.2 Temporal Reproducibility and Contribution Beyond Previous NHANES Studies

Similar estimates across NHANES survey periods support within‐survey temporal reproducibility. This finding extends earlier cross‐sectional and spline analyses without providing an independent US population replication. Li and colleagues used 27 components and excluded laxative and antibiotic users (Li, Chen, et al. 2024). Wang and colleagues used 25 components and included symptom classification and mediation analyses (Wang et al. 2025). The latter article was authored by Haiyang Wang and colleagues, not by this study's corresponding author, Honggang Wang. Both previous studies and our operational outcome include mucus, liquid or solid leakage. Table S1 compares their samples, exposure construction, outcomes and analyses with ours. The present study adds period‐specific analyses, a partially harmonized external comparison and prospective outcomes in two additional settings. These analyses address whether the concurrent association extends across dietary measurements and to subsequent symptoms. Availability of the relevant bowel‐health items determined the NHANES study period (National Center for Health Statistics 2007). Changes in diets, food formulation, food access, and socioeconomic circumstances since 2005–2010 may limit contemporary generalizability. Comparisons with other countries do not update the historical US association to the present day.

### 4.3 Dietary Measurement and the ELSA Comparison

The common 15‐component score was associated with concurrent bowel‐control problems in ELSA. Matching components reduces one source of heterogeneity, but differences in dietary instruments, population ages and outcomes remain. ELSA used the online Oxford WebQ in an aging cohort that included younger partners. NHANES used interviewer‐administered recall in adults aged at least 20 years. Online participation, recall error and nutrient distributions may affect both score distributions and estimated associations. ELSA assessed bowel‐control problems over 12 months, whereas NHANES measured frequency‐defined mucus or stool leakage over 30 days. These differences can alter prevalence and case composition, so similar estimates do not establish measurement equivalence. The uncertain carotene mapping adds another limitation. Excluding this component assesses sensitivity to the mapping, without validating equivalence between instruments. Weighting attenuated the ELSA estimate. With the core Wave 9 weight multiplied by nutrition‐participation IPW, the OR was 1.088 (95% CI 0.998–1.187). The estimate remained positive, but the interval included the null. Participation and attrition IPWs address observed selection and depend on measured predictors, model specification and adequate overlap. They do not remove unmeasured selection or recreate nutrition‐specific sampling and calibration weights. The findings therefore should not be treated as population‐representative estimates for England.

### 4.4 Prospective Findings and Temporal Interpretation

Baseline dietary scores showed no clear associations with incident outcomes in ELSA or CLHLS. Cumulative incident ORs were 0.974 (95% CI 0.902–1.051) and 1.044 (0.920–1.184), respectively, using different exposure units and outcomes. These findings limit a prospective interpretation of the concurrent associations. However, statistical significance in concurrent analyses and its absence in incident analyses do not establish a difference between the associations. Reverse causation is one plausible explanation for the concurrent findings. Older women with FI have described avoiding foods, changing preparation methods, and modifying meal size or frequency to manage symptoms (Andy et al. 2020). Avoidance of nutrient‐rich foods could raise an inflammatory score, whereas avoidance of fatty foods or alcohol could lower it. Diet recorded after symptom onset may therefore partly reflect symptom management, although our data do not establish this explanation. Longitudinal measurement and selection also require consideration. A baseline dietary measure may poorly capture changes over subsequent years, and exposure error can obscure associations. Intermittent symptoms may be missed between waves, while competing mortality and nonresponse alter the population observed at follow‐up. Weighting and imputation cannot fully resolve these problems. The confidence intervals describe remaining uncertainty rather than establish equivalence to no association. Differences in dietary assessment, outcome definition, follow‐up, and population composition may help explain the prospective association previously reported in older women (Yang et al. 2023). Thus, the present findings should not be generalized to every diet or pathway to incontinence.

### 4.5 CLHLS Extension and Functional Context

The CLHLS analysis extends the question to older Chinese adults using a 13‐food directional score and combined urinary/bowel continence impairment. It does not directly validate DII or an FI‐specific association. Food‐frequency categories do not reconstruct nutrient quantities, energy intake, or portion sizes. The combined outcome includes catheter assistance or inability to control, making urinary disease, disability, and care dependence relevant to interpretation. Its OR per score standard deviation cannot be ranked against an OR per DII unit as evidence of a larger biological effect. The score assigns lower values to more frequent consumption of several plant foods and selected nutrient‐dense foods. Meat, salt‐preserved vegetables, and sugar contribute in the opposite direction. These directions are assumptions, not established inflammatory effects of every food in this population. The categories do not distinguish meat type, dairy formulation, or tea preparation. Eggs and dairy may reflect dietary adequacy or access, while tea may accompany different social and dietary patterns. The alternative 10‐item score tests dependence on these choices; its association does not biologically validate the score. Functional and socioeconomic circumstances offer additional explanations. Frailty, impaired mobility, cognitive decline, and disability can limit shopping, cooking, eating, and toileting. Medication use and comorbidity affect continence, appetite, and food choices, while caregiver support and socioeconomic circumstances shape food access. Food insecurity has been associated with FI in US women (Okada et al. 2023). The available covariates capture only part of this potential confounding. Both complete‐case and imputed cognition‐adjusted estimates were attenuated, with confidence intervals including the null. Changes in sample composition and missingness treatment prevent attributing this attenuation solely to cognition. Excluding proxy‐assisted interviews left few events, illustrating the trade‐off between information source and coverage of more impaired participants.

### 4.6 Implications for Nutritional Epidemiology and Assessment

These comparisons highlight the importance of distinguishing diet recorded alongside symptoms from diet measured before symptom onset. When dietary history is assessed, the timing of restrictions and other dietary changes relative to symptoms is important. Usual intake, preparation methods, stool characteristics, medication use, functional status and food access provide context for interpreting dietary scores (Figure 6d). This assessment framework is not a validated clinical pathway. The findings do not establish that lowering DII improves continence. Benefits from screening or dietary modification require separate evaluation in prediction or intervention studies.

### 4.7 Strengths, Limitations, and Future Research

Strengths include complex‐survey analysis in NHANES, assessment of period‐specific reproducibility and dietary construction, and prospective analyses in two additional settings. Dietary measurement differences and incompletely measured functional and socioeconomic factors limit interpretation across populations. CLHLS oversamples the oldest‐old and is subject to survival and response selection. Several statistical limitations remain. ELSA nutrition‐specific calibration weights were unavailable, and CLHLS sampling clusters and strata were not reconstructed. Participant‐level robust variance therefore does not provide full complex‐survey inference for CLHLS. Estimated selection weights were treated as fixed, without propagating their estimation uncertainty. Hot‐deck imputation depends on donor and missingness assumptions and does not guarantee unbiased inference. Repeated‐measures associations likewise do not establish a causal effect of changing diet. Future studies should collect repeated dietary measurements, the timing and reasons for dietary restrictions, and separate urinary and fecal outcomes. Validated symptom measures and continence‐related quality‐of‐life measures would better characterize the outcome (Rockwood et al. 1999; Rockwood et al. 2000). Prospective designs should also measure functional status, food access, medication changes, and competing mortality. Intervention studies should test specified dietary changes and monitor nutritional adequacy, rather than assume that lowering a composite inflammatory score improves continence.

## 5 Conclusion

Higher dietary scores were associated with existing continence‐related symptoms in the main analyses, although some associations were sensitive to weighting and cognitive adjustment. Baseline scores showed no clear associations with incident outcomes in ELSA or CLHLS. These findings extend cross‐sectional evidence while limiting its interpretation as evidence for prevention. Differences in dietary measures and outcome definitions preclude treating the three populations as direct replications.

## Author Contributions

**Mengmeng Jiang:** investigation, data curation, writing – original draft, methodology. **Mengshi Zhong:** data curation, writing – original draft. **Juan Yu:** data curation, writing – original draft. **Jialing Zhang:** data curation, writing – original draft. **Honggang Wang:** conceptualization, investigation, funding acquisition, writing – review and editing, data curation.

## Funding

The Scientific Research and Innovation Team of Huai'an First People's Hospital (YCT202305).

## Ethics Statement

Ethical Review: NHANES 2005–2006 was approved by the National Center for Health Statistics Research Ethics Review Board under protocol 2005–06, with continuations covering 2007–2008 and 2009–2010 (National Center for Health Statistics 2026). ELSA Waves 9, 10, and 11 received approval from the South Central‐Berkshire Research Ethics Committee under references 17/SC/0588, 21/SC/0030, and 23/SC/0112, respectively (English Longitudinal Study of Ageing n.d.‐a). Published CLHLS reports describe approval by the research ethics committees of Peking University and Duke University (Zeng et al. 2017). A subsequent report covering CLHLS waves through 2018 specifies approval by the Biomedical Ethics Committee of Peking University (Li, Wang, et al. 2024). The present study was a secondary analysis of de‐identified survey data and involved no additional participant recruitment, direct participant contact, or intervention. No separate institutional ethics approval or formal exemption determination was obtained for this secondary analysis. Informed Consent: NHANES participants provided documented consent through the survey consent process. ELSA obtained consent according to its wave‐specific protocols and consent documentation (English Longitudinal Study of Ageing n.d.‐b). For CLHLS, the published report covering waves through 2018 states that participants or their proxy respondents signed informed consent (Li, Wang, et al. 2024). No participants were recontacted to obtain additional consent for the present secondary analysis.

## Conflicts of Interest

The authors declare no conflicts of interest.

## Supporting information


**Table S1:** Comparison with earlier NHANES studies.
**Table S2:** Measurement and interpretation across populations.
**Table S3:** Additional measurement sensitivities.

## Data Availability

NHANES data are publicly available from the United States National Center for Health Statistics. ELSA Waves 0–11 are available through the UK Data Service under registered access (Study 5050). CLHLS 2008–2018 data are available under the data‐use agreement of the Peking University Open Research Data Platform. NHANES: https://www.cdc.gov/nchs/nhanes/. ELSA: UK Data Service, Study 5050. CLHLS: https://doi.org/10.18170/DVN/WBO7LK. Access to ELSA and CLHLS is subject to the providers' registration and data‐use conditions. Participant‐level records are not redistributed with this article. Additional details of dietary score construction are provided in the Supporting Information.
