# Supplementary material for: Dietary Inflammatory Potential and Continence‐Related Symptoms: Cross‐Sectional and Longitudinal Analyses of Three Population Studies
Source: Food Sci Nutr. 2026 Oct 9;14(10):e72477. doi: 10.1002/fsn3.72477 (PMC13653072; doi:10.1002/fsn3.72477)
Supplement: Supplementary file 1 [file FSN3-14-e72477-s001.docx]

# Supporting Information

# Table S1 Comparison with earlier NHANES studies

| **Feature** | **Li et al. (2024)** | **Wang et al. (2025)** | **Current study** |
| --- | --- | --- | --- |
| Data | NHANES 2005-2010 | NHANES 2005-2010 | Same NHANES cycles plus ELSA and CLHLS |
| Analyzed NHANES sample | 11,747; ages 20-85 | 9,377; age ≥20 | 13,385; age ≥20; primary complete-case model |
| Dietary construction | 27 components; two-day recall reported; dietaryindex package | 25 components | 25-component primary index; day-1 recall; 15-component comparison; two-day sensitivity |
| Primary outcome | Mucus, liquid or solid leakage in prior month | Mucus, liquid or solid leakage in prior month | At least monthly mucus, liquid or solid leakage in prior 30 days; same broad operational types |
| Selection and adjustment | Excludes laxative/antibiotic users; demographic, lifestyle and health adjustments | Complete cases; demographic, lifestyle and comorbidity adjustments | Core and extended models specified in Methods; different selection rules |
| Analysis focus | Weighted logistic models, splines, subgroups | Logistic models, splines, dietary feature selection, classification and mediation | Period-specific reproducibility, dietary sensitivity, partial harmonization, prospective and repeated-measures analyses |
| Contribution and overlap | Previously reported cross-sectional association | Previously reported cross-sectional association and classification approach | NHANES is overlapping source data; independent contribution is the cross-setting and prospective interpretation, not first discovery |

Li et al. (2024), doi:10.3389/fnut.2024.1364835; Wang et al. (2025), doi:10.3389/fnut.2025.1547406. Details summarize the published methods; no participant-level overlap was calculated.

# Table S2 Measurement and interpretation across populations

| **Feature** | **NHANES** | **ELSA** | **CLHLS** |
| --- | --- | --- | --- |
| Population | US adults ≥20 | England ageing cohort plus younger partners; age ≥50 sensitivity | Chinese adults ≥65; oldest-old oversampled |
| Time structure | 2005-2006, 2007-2008, 2009-2010 repeated cross-sections | Wave 9 linked to Waves 10 and 11 | 2008 linked to 2011, 2014 and 2018 |
| Diet instrument | Interviewer-administered 24-hour recall | Online Oxford WebQ 24-hour questionnaire | Food-frequency items |
| Exposure unit | 25-component index; 15-component comparison; per unit | Common 15-component index; per unit | 13-food score; per baseline standard deviation |
| Outcome | Monthly mucus, liquid or solid leakage in prior 30 days | Bowel-control problem in prior 12 months | Combined urinary/bowel control; includes catheter assistance |
| Role | Within-survey temporal reproducibility | Partially harmonized external comparison and prospective boundary | Conceptually aligned extension and prospective boundary |
| Interpretation constraint | Historical US setting; concurrent diet and symptoms | Different instrument, outcome and selection; nutrition-specific calibration weight unavailable; core weight tested | Different construct, scale and outcome; functional/cognitive confounding |

Differences in odds ratios may reflect measurement, age, selection and adjustment as well as association differences. No pooled cross-cohort effect is estimated.

# Table S3 Additional measurement sensitivities

| **Analysis** | **N/events** | **OR** | **95% CI** | **P** |
| --- | --- | --- | --- | --- |
| NHANES without carotene | 13,385/1,213 | 1.150 | 1.066-1.240 | <0.001 |
| ELSA age ≥50 concurrent | 4,110/644 | 1.138 | 1.068-1.213 | <0.001 |
| ELSA age ≥50 incident | 2,818/427 | 0.973 | 0.901-1.051 | 0.484 |
| ELSA without carotene concurrent | 4,141/646 | 1.160 | 1.072-1.254 | <0.001 |
| ELSA without carotene incident | 2,837/430 | 0.962 | 0.875-1.058 | 0.427 |

ORs are per one score unit. The 14-component models exclude the uncertain carotene mapping in both cohorts. These sensitivity analyses do not calibrate instruments, food composition or outcome definitions. Age restriction is specific to ELSA. These age-restricted and 14-component analyses were added during revision; they are supplementary checks rather than prespecified primary analyses.

# Supplementary Methods

## Dietary score construction

Each term uses z=(intake−reference mean)/reference SD, centered percentile 2Φ(z)−1, then multiplication by its effect score and summation. All required terms must be observed. The original source label for ELSA carotene does not prove beta-carotene equivalence; the 14-component sensitivity excludes it. NHANES vitamin A uses retinol + beta-carotene/6 + alpha-carotene/12 + beta-cryptoxanthin/12. ELSA averages available dietary days; NHANES two-day scores average intakes before scoring.

## ELSA coding and selection models

Marital status uses dimarr codes 2 and 3 (married, remarried, or in a registered civil partnership), versus other valid categories 1, 4, 5 and 6. Missing/refused categories remain missing; this grouping does not identify actual cohabitation. Nutrition-selection logistic models use age, sex, ethnicity, education, marital status, smoking, activity, diabetes, stroke, cancer and depression. Follow-up response models add baseline dietary score among participants without baseline symptoms. Propensities are clipped to 0.01-0.99. Observed-case stabilized weights use marginal response divided by predicted response and are truncated at observed 1st/99th percentiles. Follow-up sensitivities multiply nutrition-selection and wave-response weights. The concurrent combined-weight sensitivity multiplies nutrition IPW by positive core w9xwgt. No nutrition-specific sampling/calibration weight is reconstructed.

## Imputation and repeated measures

ELSA core covariates were imputed in 20 datasets with seeds 20260805-20260824. Education is sampled as one category before deriving dummy variables. Donors are observed for each target variable, with at least five within sex, age decade, outcome and score-quartile strata; fallback uses sex/age, sex, then all observed donors. CLHLS uses the same hierarchical strata for core covariates plus MMSE with seeds 1001-1020. Exposure and outcome are not imputed. Rubin pooling combines within- and between-imputation variance and uses t-based intervals. This variable-wise hot-deck procedure does not guarantee preservation of the full multivariate covariate distribution. ELSA all-wave GEE uses baseline score, wave indicators and score-by-wave terms. Its incident discrete-time GEE censors observed rows after first reported symptoms. CLHLS four-wave GEE uses time-varying food scores, baseline covariates and baseline weights; it estimates concurrent associations, not prediction from baseline diet.

## CLHLS weights and uncertainty

CLHLS response models use baseline score and the core covariates listed in Section 2.7, with propensities clipped to 0.02–0.98. Observed-case stabilized response weights are truncated at 1st/99th percentiles and multiplied by baseline sampling weights. For independent-person weighted logistic models, A=Σwᵢμᵢ(1−μᵢ)xᵢxᵢᵀ and Uᵢ=wᵢxᵢ(yᵢ−μᵢ); covariance is A⁻¹(ΣUᵢUᵢᵀ)A⁻¹. Normalizing weights to mean one leaves coefficients and this covariance invariant. A real-data implementation check agreed with independent-person GEE to below 10⁻⁷ and was invariant to tenfold weight rescaling. Sampling-cluster/stratum information is not reconstructed and estimated-IPW uncertainty is not propagated. These limitations preclude claiming fully design-based CLHLS inference.

## Spline specifications and missing values

NHANES categorical blanks were treated as missing before complete-case selection. NHANES restricted cubic splines used knots at the weighted 5th, 35th, 65th and 95th percentiles, with the weighted median as reference. ELSA and CLHLS used the unweighted marginal 5th, 35th, 65th and 95th percentiles and median references. ELSA used a full-rank centered spline with likelihood-ratio testing of nonlinearity. CLHLS used a linear term and two nonlinear terms, with a robust Wald test of the nonlinear terms. These specifications concern association shape, not predictive validation. Participation and response weights were stabilized and truncated at the observed 1st and 99th percentiles, with cohort-specific propensity clipping as described above. The available ELSA core weight is w9xwgt. Covariance calculations treat estimated selection weights as fixed and therefore do not propagate the uncertainty in fitting the weight models. Cumulative incident outcomes identify any reported problem at an available follow-up among baseline-negative participants. Participants with at least one observed negative follow-up and no observed positive report are coded negative, even if another wave is missing. These analyses do not establish symptom-free survival throughout follow-up. They use neither exact onset times nor a common observed follow-up duration. The ELSA incident GEE omits missing waves and stops after the first observed positive report; it therefore concerns first observed reporting, not necessarily first biological onset.
